# Non-invasive monitoring allograft rejection by simultaneous cellular and functional cardiac MRI

**DOI:** 10.1186/1532-429X-11-S1-O79

**Published:** 2009-01-28

**Authors:** Yijen L Wu, Qing Ye, Kazuya Sato, Lesley M Foley, T Kevin Hitchens, Chien Ho

**Affiliations:** Pittsburgh NMR Center for Biomedical Research, Carnegie Mellon University, Pittsburgh, PA USA

**Keywords:** Heart Transplantation, Allograft Rejection, Acute Allograft Rejection, Cardiac Allograft Rejection, DANTE Sequence

## Inroduction

The current gold standard for diagnosing and staging rejection after organ transplantation is biopsy, which is not only invasive, but is also prone to sampling errors. The regimen for treating acute rejection after heart transplantation varies among treatment centers, partly due to lack of sensitive and reliable indices for assessing the status of myocardial rejection. The goal of this study is to establish sensitive and reliable indices using cellular and functional MRI for non-invasive detection of acute cardiac allograft rejection after heart transplantation. Using a rodent model of cardiac transplantation, we monitor both immune cell infiltration and cardiac dysfunction resulting from rejection in the same imaging session. Immune cells, mainly monocytes and macrophages, are labeled *in situ* with dextran-coated ultra-small superparamagnetic iron oxide (USPIO) nano-particles. T_2_*-weighted MRI and strain analysis of tagged MRI are used to correlate immune-cell infiltration and ventricular function with rejection grades.

## Methods

### 1. Animal model

An abdominal heterotopic working heart and lung transplantation model with DA to BN transplantation rat pairs was used. The transplanted hearts exhibit cardiac outputs and ventricular pressure similar to those of native hearts. In this model, mild (Grade 1A or B) rejection develops by post-operation day (POD) 2.5–3.5, Grade 2 rejection develops on POD 4.5–5.5, and moderate to the severe (Grade 3A) rejection develops after POD 6–7.

### 2. MRI methods

ECG and respiration gated T_2_*-weighted cine imaging on Bruker AVANCE 4.7-T system was used for *in-vivo* imaging with an in-plane resolution of 156 μm. Tagged MRI is achieved with a modified DANTE sequence. Strains were analyzed by the HARP method with software obtained from *Diagnosoft, Inc*.

### 3. Iron-oxide particle labeling

Immune cells, mostly macrophages, are labeled *in situ* by direct intravenous injection of USPIO particles 1 day prior to MRI scans.

## Results

**Figure 1:**
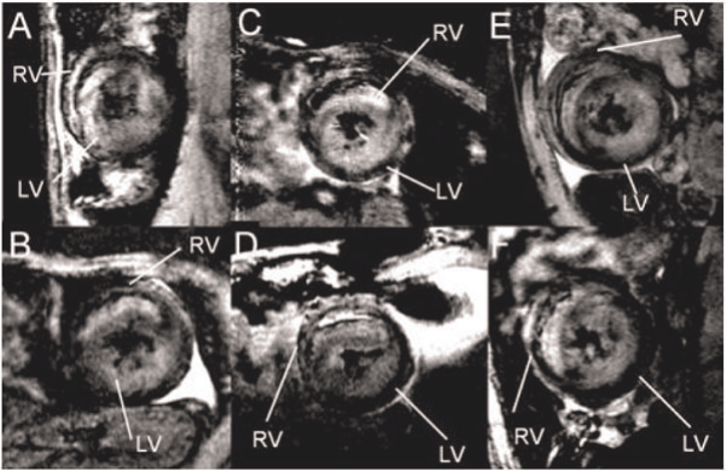
T2*-weighted ***in vivo*** **MRI for different allografts after USPIO administration**. Areas with UPSIO-labeled macrophage infiltration show signal loss in both LV and RV.

Immune cells, mainly monocytes and macrophages, take-up circulating USPIO particles by endocytosis. Foci of labeled immune cells in the rejecting graft are observed by regions of hypointensity in T_2_*-weighted images (Fig. 1). The observed immune-cell infiltration is heterogeneous, thus it is not surprising to have incidences of false-negative results with biopsy. Monitoring immune-cell infiltration with MRI is not only non-invasive, but also provides whole-volume 3D perspective of rejection.

**Figure 2:**
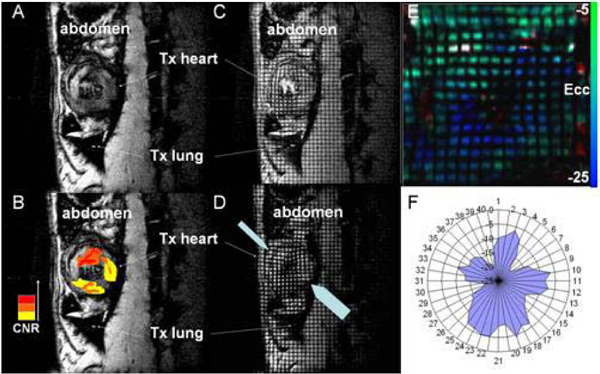
(A) T2*-weighted MRI for an allograft after USPIO administration. (B) Pseudo-coloring of different contrast-to-noise ratios (CNR) for easier visualization. (C, D) Tagged MRI of the same allograft heart at ED (C) and ES (D). The block arrows point to regions with compromised contractile functions. (E) Colored Ecc strain map of the allograft. (F) Ecc stran values of 48 probe-points throughout LV.

To discern if the heterogeneity of rejection is manifested in cardiac function, tagged MRI is used to monitor regional ventricular contractile function. Although global function may appear normal, the rejected allograft may show local hypokinesis (Fig. 2D). Strain analysis by HARP was used to quantify the local contractile function. Regions with compromised strains (Fig. 2E &2F) largely correlate with the areas of higher macrophage infiltration.

**Figure 3:**
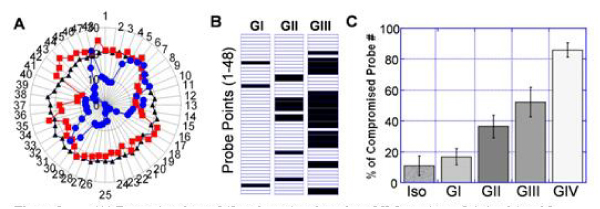
**(A) Ecc strain values of 48 probe-points throughout LV for an isograft (triangle) and 2 allografts with Grade II (square) and Grade IV (circle) rejection**. (B) Status of probe-points for 3 allografts with different rejection grades. Compromised probe-points are black, whereas the normal probe-points are left blank. (C) Degrees of compromised probe-points for different rejection grades.

For easier visualization of the regions with compromised strains, 48 probe-points were placed evenly throughout LV, starting at the anterior intercept of LV and RV (Fig. 2F & Fig. 3A). The probe-point is considered "compromised" if the strain value of the particular location deviats from the mean isograft values by more than 1 standard deviation; otherwise the probe-point is scored as "normal" (Fig. 3B). The number of compromised probe-points is well correlated with rejection grades (Fig. 3C), which can potentially be useful clinical index for rejection.

## Conclusion

In our model, acute allograft rejection after heart transplantation is spatially heterogeneous, which is manifested in both immune-cell infiltration and ventricular function. Cardiac MRI is both non-invasive and provides 3D, whole-heart perspective of rejection status, which potentially allows more reliable detection of acute allograft rejection.

